# High Histone Deacetylase 2/3 Expression in Non-Functioning Pituitary Tumors

**DOI:** 10.3389/fonc.2022.875122

**Published:** 2022-05-13

**Authors:** Wenxiu Zhao, Xiaobin Jiang, Karrin Weisenthal, Jun Ma, Erin M. Botticelli, Yunli Zhou, E. Tessa Hedley-Whyte, Baiyao Wang, Brooke Swearingen, Roy J. Soberman, Anne Klibanski, Xun Zhang

**Affiliations:** ^1^ Neuroendocrine Unit, Massachusetts General Hospital and Harvard Medical School, Boston, MA, United States; ^2^ Neuropathology Unit, Massachusetts General Hospital and Harvard Medical School, Boston, MA, United States; ^3^ Neurosurgical Service, Massachusetts General Hospital and Harvard Medical School, Boston, MA, United States; ^4^ Nephrology Division, Massachusetts General Hospital and Harvard Medical School, Boston, MA, United States

**Keywords:** human clinically non-functioning pituitary adenomas, class I histone deacetylases, growth suppression, epigenetic modifications, regulation of tumor suppressor expressions

## Abstract

Epigenetic modification of chromatin is involved in non-malignant pituitary neoplasia by causing abnormal expression of tumor suppressors and oncogenes. These changes are potentially reversible, suggesting the possibility of targeting tumor cells by restoring the expression of epigenetically silenced tumor suppressors. The role of the histone deacetylase (HDAC) family in pituitary tumorigenesis is not known. We report that HDAC2 and 3, Class I HDAC members, are highly expressed in clinically non-functioning pituitary adenomas (NFPAs) compared to normal pituitary (NP) samples as determined by RT-PCR and immunohistochemical staining (IHC). Treatment of a human NFPA derived folliculostellate cell line, PDFS, with the HDAC3 inhibitor RGFP966 for 96 hours resulted in inhibition of cell proliferation by 70%. Furthermore, the combination of RGFP966 with a methyltransferase/DNMT inhibitor, 5’-aza-2’-deoxycytidine, led to the restoration of the expression of several tumor suppressor genes, including STAT1, P16, PTEN, and the large non-coding RNA tumor suppressor MEG3, in PDFS cells. Our data support the hypothesis that both histone modification and DNA methylation are involved in the pathogenesis of human NFPAs and suggest that targeting HDACs and DNA methylation can be incorporated into future therapies.

## Introduction

Pituitary adenomas are one of the most common intracranial neoplasms, comprising approximately 10% of all surgically resected intracranial tumors, with a prevalence of 22.5% in the general population (1). Cross-sectional studies have shown that the prevalence of symptomatic pituitary adenomas ranges from 7.76 in 10,000 to 9.4 per 10,000 individuals (2, 3). Non-functioning pituitary adenomas (NFPAs) that do not secrete hormones are common and are mainly derived from gonadotroph cells. The majority of these tumors are benign. However, patients can experience increased morbidity due to mass effect, causing neurologic complications, and a subset are aggressive (4–6). These tumors are routinely treated with surgery and radiation. However, a subset of these requires additional therapies, such as temozolomide. The vast majority of these tumors escape from these temozolomide, and additional options are needed (7). There are no effective or FDA-approved pharmacologic therapies to treat these tumors. Therefore, it is critical to identify novel targets in NFPAs for the future development of medical therapy.

In sporadic pituitary adenomas, genetic mutations of oncogenes or tumor suppressors are extremely rare, and epigenetic dysregulation is the major factor dictating the expression of multiple tumor suppressor genes (8, 9). Studies have focused on how aberrant hypermethylation of CpG islands within promoters contributes to the inactivation of several important genes, including *RB1*, *CDKN1A(P21)*, *CDKN2A*, *CDKN2B*, *GADD45γ*, and *MEG3* (9). The role of histone modification, though firmly linked to the genesis of other tumors such as breast and colorectal cancer, has not been explored in pituitary tumorigenesis, especially in NFPAs.

Histone deacetylases (HDACs) are responsible for removal of acetyl groups from specific lysine residues located within the protein tails of histones (10, 11) as well as many non-histone proteins, including transcription factors (10, 11). The family of Class I HDAC includes HDAC1, HDAC2, HDAC3, and HDAC8. HDAC2 and HDAC3 are important for control of cell cycle regulation, cardiac function, and neural cell regulation (12, 13). Abnormal expression of HDAC1 has been reported in breast, gastric, colon, liver, renal and prostate carcinomas (14–16). High levels of HDAC2 expression have been shown in cervical, gastric (17, 18), breast, and other cancer types (16, 19). In addition, high levels of HDAC3 expression have been reported in colon cancer (20). Furthermore, the expression of HDAC1 has been reported to correlate with the progression and prognosis of gastrointestinal malignancies (21, 22); and HDAC3 also has been suggested as a prognostic marker in gastric (21), colorectal (23), and pancreatic cancer (24). *In vitro* experiments have shown that high levels of HDAC1 expression promotes migration and invasion of gallbladder (25) and breast tumor cell lines (26). Similarly, overexpression of HDAC2 has been shown to promote the migration and invasion of non-small cell lung cancer cell lines (27), and overexpression of HDAC3 has been reported to promote the proliferation of cholangiocarcinoma and pancreatic cancer cell lines (28, 29). Therefore, there is compelling evidence that high levels of HDAC1, 2 and 3 expression is implicated in the pathogenesis of several malignant tumors. However, there is no reported link between Class I HDAC and benign tumors such as NFPAs.

Because there is no reliably effective medical therapy for human clinically non-functioning pituitary adenomas, we focused our study on this type of pituitary tumor. We hypothesized that Class I HDAC RNA levels would be higher in NFPAs compared with normal human pituitary samples. We found that HDAC3 and HDAC2 RNA, but not HDAC1 RNA, were significantly higher in NFPAs compared to normal human pituitaries; increased HDAC3 protein expression was also confirmed by immunohistochemical (IHC) staining. The HDAC3-specific inhibitor RGFP966 suppressed PFDS [a folliculostellate cell line derived from a human NFPA (30),] cell proliferation by 70% at 96 hours after treatment. Treatment of PDFS cells with RGFP966 restored expression of MEG3, a long non-coding RNA tumor suppressor, whose expression is lost in NFPAs but not in hormone secreting pituitary adenomas (9). Furthermore, the combination of the DNA methyltransferase (DNMT) inhibitor 5’-aza-2’-deoxycytidine (5’-AZA) with RGFP966 further enhanced MEG3 expression and also activated expression of several well-known tumor suppressors including P16, PTEN, and STAT1. Therefore, we demonstrate that suppression of histone deacetylation and DNA methylation restores epigenetically silenced tumor suppressors such as MEG3, P16, PTEN and STAT1, resulting in inhibition of tumor cell growth. This study suggests HDAC3 inhibition may be a potential therapeutic approach for NFPAs.

## Material and Methods

### Tissue and Tumor Samples

Twenty-two human NFPA, as well as six GH-secreting, six ACTH-secreting, and six PRL-secreting human pituitary adenoma surgical samples were obtained at Massachusetts General Hospital and used for RT-PCR analysis. The diagnosis of NFPA was established by clinical, biochemical, and radiological findings and was confirmed by immunohistochemistry after surgery. Human pituitary tumors were collected in 0.9% saline after transsphenoidal surgery and immediately frozen in liquid nitrogen before analysis. Eleven normal human pituitary glands were obtained 2-16 hours postmortem from the Harvard Tissue Resource Center (Belmont, MA). This study was approved by the Partners Human Research Committee. Immunohistochemical staining on pituitary tumor slides was performed by Pathology Department of Massachusetts General Hospital on formalin-fixed, paraffin embedded sections for FSHβ, LHβ, TSHβ, prolactin, GH, ACTH, and glycoprotein hormone α-subunit, to confirm the tumor identities.

### RNA Extraction, cDNA Synthesis and Quantitative Real-Time PCR

Total RNA was isolated using the RNeasy Mini Kit (QIAGEN, USA) according to the manufacturer’s instructions. Extracted RNA samples were treated with DNase I to remove potential DNA contamination. One microgram of total RNA was subjected to reverse transcription using a ProtoScript^®^M-MuLV first-strand cDNA synthesis kit (New England Bio-Labs, Ipswich, MA, USA). All PCR reactions were performed in triplicate with an Applied Biosystems^®^ 7500 Fast Thermocycler (Foster City, CA, USA) with the following protocol: 50°C for 2 minutes, 95°C for 5 minutes, followed by 40 cycles of 95°C for 30 seconds and 60°C for 1 minute. Melting curve analysis was performed and a no-template control was included in every qPCR using Invitrogen SYBR^®^PCR Master Mix (Thermo Fisher, Waltham, MA, USA). GADPH was used as internal controls. Expression levels for tested genes were calculated using the formula 2^-△Ct^. The fold changes were determined by 2^-△△Ct^. The relative tumor expression levels for genes were calculated by normalizing the value from the tumor against the mean average of values from the 11 normal pituitaries. Primers used in this study are listed in **Table S1**.

### Immunohistochemistry Staining

A tissue microarray (TMA) containing 12 human NFPAs and 4 normal human pituitaries, which were distinct from the samples used in qRT-PCR, was constructed by the Department of Pathology Service Core Facility of Massachusetts General Hospital, and 4 μm sections were prepared from the TMA. Immunohistochemical staining was performed as previously described (25), with mouse monoclonal anti-HDAC3 (Abcam, ab219376, Cambridge, MA, USA) at 1:1000 dilution, or anti-HDAC2 (Abcam, ab16032) at 1:500 dilution. HDAC3/HDAC2 staining was considered positive only when showing definitive staining of tumor nuclei. Replacement of a primary antibody with PBS served as the negative control. Positive HDAC3/HDAC2 staining was assessed microscopically in 10 high-powered fields (×200) and quantified on the basis of the color intensity of photos (40x) converted to CYMK and split into green/red/yellow/blue channels. To collect data,100 nuclei were selected and averaged using CellProfiler3.0.0 software (Broad institute, Cambridge, MA, USA, http://cellprofiler.org/).

### PDFS Cell Culture and Treatment With Inhibitors

The pituitary tumor-derived folliculostellate cell (PDFS) line (30) was maintained in DMEM (Life Technologies) supplemented with 10% FBS, 1% NEAA and PSG at 37°C with 10% CO_2_. DNA methyltransferase inhibitor 5’-aza-2’-deoxycytidine (5’-AZA) was purchased from Sigma-Aldrich (Allentown, PA, USA). The HDAC3-specific inhibitor RGFP966 was purchased from Selleckchem (Houston, TX, USA) (31). To examine the effect of HDAC inhibition on the growth of PDFS, 1x10^6^ PDFS cells were plated on 100 mm cell culture dishes. After 24 hours, cells were treated with 10μM RGFP966 and/or 1μM 5’-AZA; with DMSO as a control. Culture medium was changed daily with fresh drug added. RNA was extracted from cells at Day 5. Cell proliferation was monitored at 24, 48, 72, and 96 hours by CCK8 Assay (see below).

### Cell Growth (CCK8 Assay)

Cell proliferation was evaluated by a CCK8 Assay (Dojindo Molecular Technologies, Rockville, MD, USA). PDFS cells were plated into a 96-well plate in triplicate, with 100 l μL cell suspension (2000 cells/well) in each well. Cells were incubated for 24 hours at 37C, with 10% CO2, before the treatment drug was added. At each time point, 10 μL of CCK-8 Solution was added to each well of the plate. The plates were further incubated for 4 hours. The absorbance at 450 nm was measured using a Versamax Tunable Microplate Reader (Molecular Devices, San Jose, CA, USA). The experiment was repeated three times.

### Statistical Analysis

Differences between quantitative parameters were expressed as mean ± SD (normal distribution) or medians with inter-quartile range (IQR). The statistical significance of gene expression was assessed by a two-tailed Student *t* tests. Associations between clinical, biological, imaging and HDAC3 expression were evaluated by Spearman’s correlations, χ^2^ tests, or Fisher’s exact tests. A p < 0.05 was considered statistically significant. All statistical analyses were performed with Prism 6 (GraphPad Software, La Jolla, CA, USA).

## Results

### Expression of HDAC2 and HDAC3 in NFPAs

**Figure 1:**
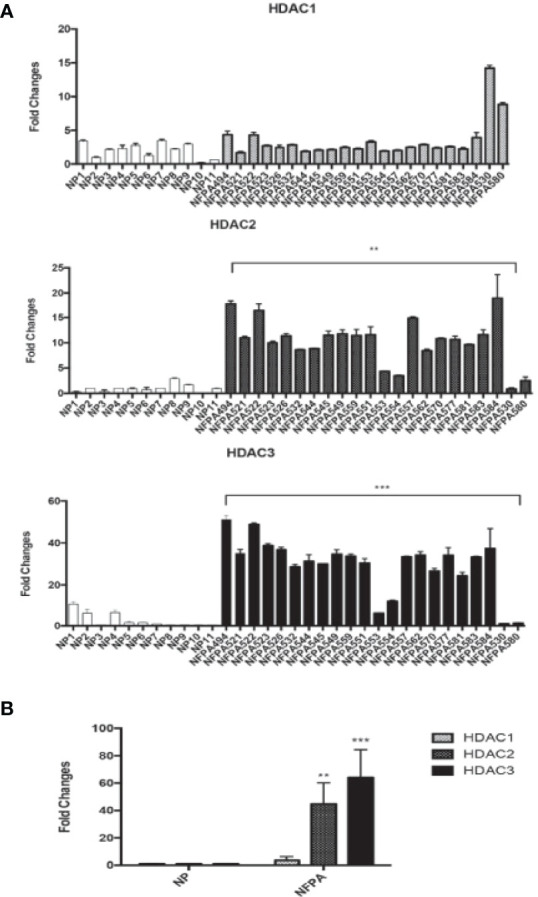
HDAC1, 2, and HDAC3 mRNA expression in human NFPAs. **(A)** HDAC1 (Upper Panel), HDAC 2 (Middle Panel), and HDAC 3 (Bottom Panel) RNA expression were determined by qRT-PCR in 22 non-functioning pituitary adenomas. **(B)** The average expression levels of HDAC1, 2, and 3 mRNA in NFPAs compared to the average level in the normal pituitaries. Fold changes were determined by comparing the HDAC levels in tumors with that in normal pituitaries (NP, n = 11), which was set as 1. NFPA, non-functioning pituitary adenoma; **P < 0.001; ***P < 0.0001.

**Figure 2:**
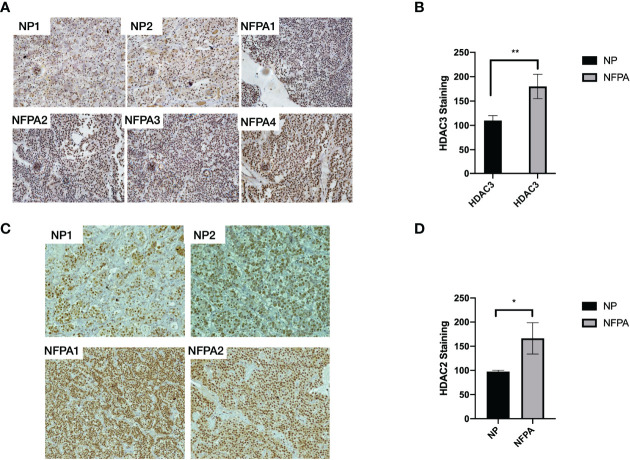
HDAC3 protein staining in normal human pituitaries and NFPAs. **(A)** Representative immunohistochemical staining images of HDAC3 protein in 2 NPs and 4 NFPAs. **(B)** HDAC3 immunostaining was quantified on the basis of color intensity of converted photos (40x) to a subtractive color model CMYK and split into green/red/yellow/blue channels. The intensities were averaged from 100 selected nuclei. **(C)** Representative immunohistochemical staining images of HDAC2 protein in 2 NPs and 2 NFPAs. **(D)** HDAC2 immunostaining was quantified on the basis of color intensity of converted photos (40x) to a subtractive color model CMYK and split into green/red/yellow/blue channels. The intensities were averaged from 100 selected nuclei. NP, normal pituitary; NFPA, non-functioning pituitary adenoma; *P < 0.05; **P < 0.001.

We detected the expression of HDAC2 and HDAC3 in 22 human NFPA specimens and 11 normal pituitary specimens by qRT-PCR. As shown in **Figure 1A**, higher HDAC2 and HDAC3 mRNA expression was observed in 82% of NFPAs. When compared to the average HDAC3 mRNA level in the normal pituitary, there was a 64-fold higher HDAC3 mRNA expression in NFPAs (**Figures 1A, B**, 64.0 ± 20.5, p < 0.0001). The average HDAC2 mRNA level in NFPAs was by 45-fold higher than in the normal pituitary samples (**Figure 1B**, 44.7 ± 15.5, p < 0.001). HDAC1 mRNA expression was also observed by qRT-PCR; but there was no significant difference in its expression levels between NFPAs and the normal pituitary samples (**Figures 1A, B**). No HDAC8 expression was detected in either NFPAs or normal pituitaries (data not shown). Immunohistochemical staining revealed higher nuclear staining of HDAC3 protein in NFPAs compared with the HDAC3 staining in normal pituitary (**Figure 2A**), The HDAC3 staining results were quantified on the basis of color intensity (**Figure 2B**). Similar results were observed for HDAC2 immunostaining (**Figures 2C, D**). These findings demonstrate the upregulation of HDAC3 and HDAC2 in NFPAs.

### HDAC2 and HDAC3 Expression in Hormone-Secreting Pituitary Adenomas

**Figure 3:**
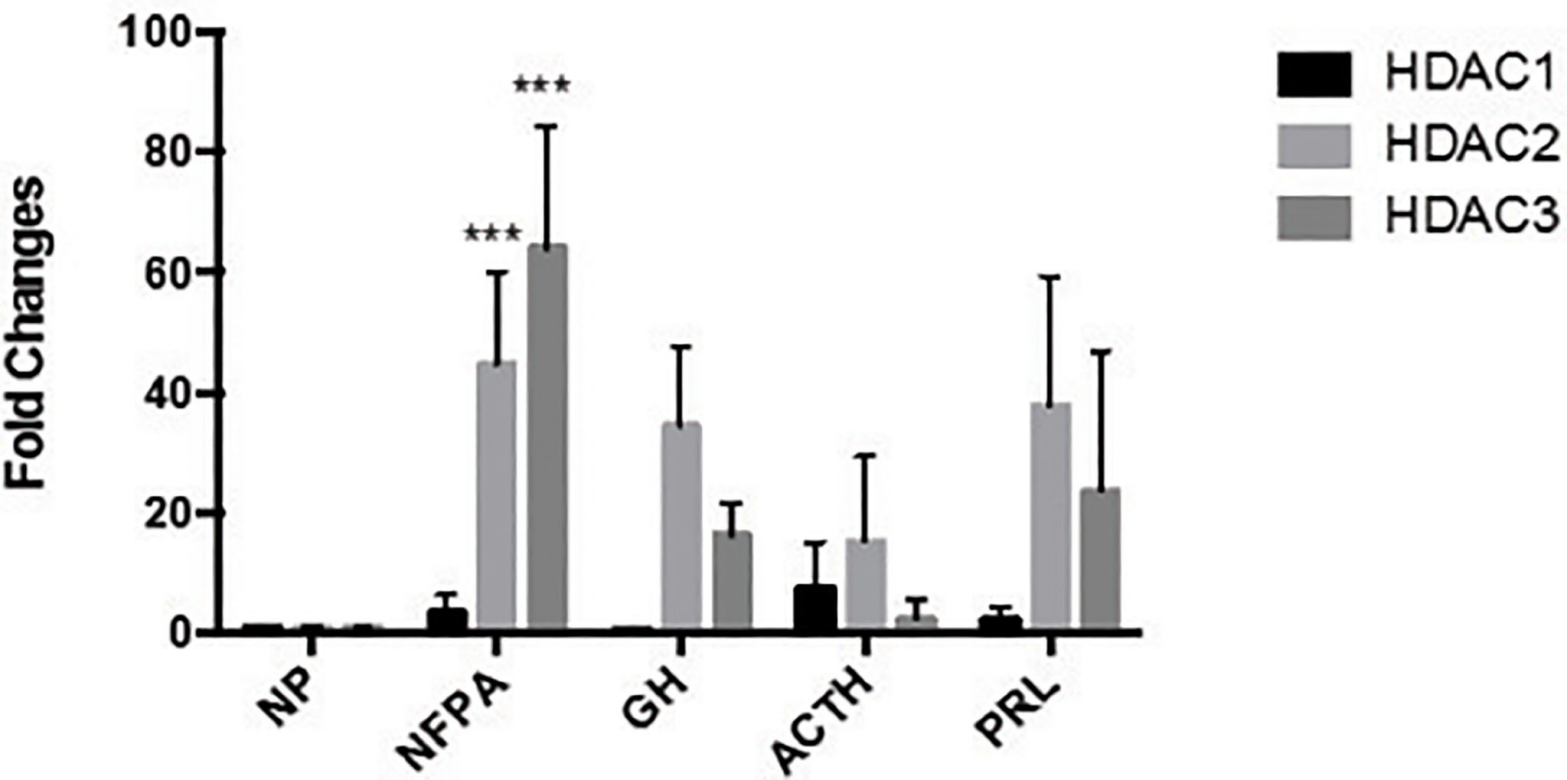
The expression of HDAC family HDAC1, HDAC2 and HDAC3 in different types of pituitary tumors. HDAC family number HDAC1, HDAC2 and HDAC3 expressed in normal pituitary (NP, n = 11) and clinically non-functioning pituitary adenomas (NFPA, n = 22), growth hormone secreting tumors (GH, n = 6), adrenocorticotropic hormone secreting tumors (ACTH, n = 6) and prolactin secreting tumors (PRL, n = 6), ***P < 0.0001.

We also examined HDAC1, HDAC2, and HDAC3 expression by qRT-PCR in six GH-secreting, six ACTH-secreting, and six PRL-secreting human pituitary adenomas. Increased mRNA expression of Class I HDAC members was also found in a limited number of hormone-secreting human pituitary adenomas (**Figure 3**), although no statistically significant difference was found in RNA expression levels between normal human pituitaries and hormone-secreting pituitary tumors. Because the increase of HDAC2 and HDAC3 levels in pituitary tumors were similar, and there is no HDAC2-specific inhibitor available, we decided to focus our subsequent studies on HDAC3.

### HDAC3 Inhibitor RGFP966 Suppressed PDFS Cell Growth

**Figure 4:**
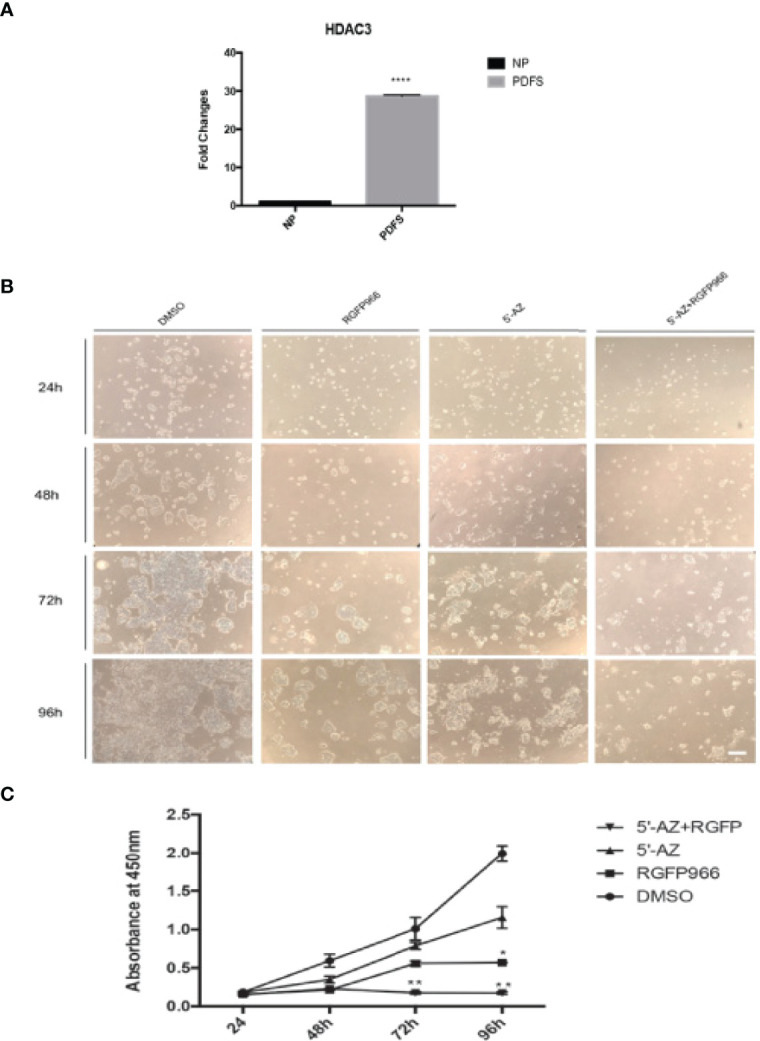
HDAC3 inhibition and DNA demethylation decreases PDFS cell proliferation. **(A)** HDAC3 expression in PDFS cells and normal pituitary. ****p < 0.00001. **(B)** PDFS treated with HDAC3 inhibitor RGFP966 (10 μM) and DNMT inhibitor 5’-AZA (1 μM), alone and in combination, at 24, 48, 72, and 96 hours. DMSO was added as a control. **(C)** Growth curves of PDFS cells as determined by CCK8 Assays. *P < 0.01; **P < 0.001; ****P < 0.00001. Scale Bar = 100μm.

Because the high expression of HDAC3 RNA and protein in NFPAs suggested that HDACs could play a role in tumorigenesis of NFPAs, we examined whether RGFP966 (31), a specific HDAC3 inhibitor, could inhibit proliferation of PDFS; which also showed very high levels of HDAC3 expression (**Figure 4A**) compared with normal pituitary. Treatment of PDFS cells with 10 μM RGFP966 suppressed cell proliferation by 64 ± 2.8%, 55.2 ± 2.3%, and 72.1 ± 1.2% (p<0.01) at 48, 72, and 96 hours, respectively, comparing with PDFS treated with DMSO (**Figure 4C**). We also found that 5’-aza-2’-deoxycytidine (5’-AZA), a demethylation reagent, was also able to suppress proliferation of PDFS, although its effect was not as strong as RGFP966. As shown in **Figures 4B, C**, treatment of PDFS cells with 1 μM of 5’-AZA suppressed cell proliferation by and 41.3 ± 2.8%, 21.9 ± 4.8%, and 42 ± 3.5% at 48, 72, and 96 hours, respectively. A synergistic effect was observed when RGFP966 was combined with 5’-AZA, suppressing PDFS proliferation by 61.4 ± 2.4%, 82 ± 4.3% (p<0.001), and 92 ± 6.1% (p<0.001) at 48, 72, and 96 hours, respectively (**Figures 4B, C**).

### Restoration of MEG3 Expression by HDAC3 Inhibition and DNA Demethylation

MEG3 is a large non-coding RNA (lncRNA) tumor suppressor whose expression is lost in NFPAs (32). PDFS cells lack expression of MEG3 due to gene silencing by DNA methylation (33, 34). To understand the molecular events related to growth suppression by RGFP966 and 5’-AZA, we examined the expression of MEG3 in PDFS cells treated with RGFP966 and 5’-AZA. As shown in **Table 1**, treatment of PDFS cells with 10 μM RGFP966 resulted in a 9-fold increase in MEG3 RNA expression compared to untreated cells. The demethylation reagent 5’-AZA (1μM) increased MEG3 RNA expression by approximately 28,000-fold. When RGFP966 and 5’-AZA were used together, MEG3 RNA expression increased by almost 140,000-fold compared to controls, confirming the synergistic effect of RGFP966 and 5’-AZA (**Table 1**).

**Table 1:** Induction of MEG3 expression by 5’-AZA and RGFP966.

Treatment	MEG3(Mean Fold Change)	SD	*P* Value
DMSO	1	0.110	
5’-AZA	28842	585.362	<0.0001
RGFP966	9	2.890	0.0084
5’-AZA+RGFP966	135512	1709.604	<0.0001

### Activation of the Retinoblastoma Pathway and Other Tumor Suppressors

**Figure 5:**
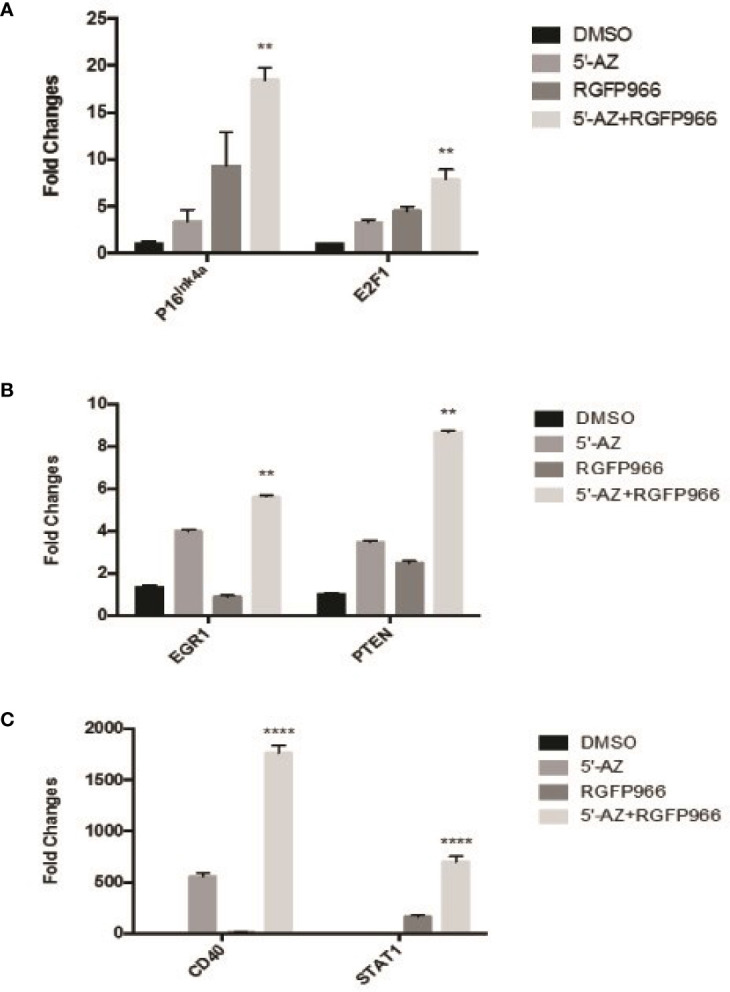
The expression of tumor suppressors induced by HDAC3 and 5’-AZA. **(A)** Induction of E2F1, P16^Ink4a^ by 5’-AZA and RGFP966. **(B, C)**: The expression of EGR1 and PTEN** (B)**, and CD40 and STAT1 **(C)** induced by 5’-AZA and RGFP966. **p < 0.001, ****p < 0.00001.

RGFP966 functioned as a strong growth suppressor, but a weak inducer for MEG3 RNA. However, the demethylation reagent 5’-AZA was a strong inducer of MEG3 expression but a weak growth suppressor (**Figure 4** and **Table 1**). These data suggest that RGFP966 may also affect expression of additional tumor suppressors. It has been reported that the signaling pathways of retinoblastoma (Rb) have been compromised in human NFPAs (9). As shown in **Figure 5A**, treatment of PDFS cells with RGFP966 significantly increased the mRNA expression of two important components of Rb pathway, p16^ink4a^ and E2F1, by 3.4- and 3.2-fold, respectively. Consistent with its strong growth suppressive function, RGFP966 was more potent than 5’-AZA in inducing p16^ink4a^ and E2F1 mRNA expression, thus activating Rb signaling pathway. Treatment of PDFS cells with RGFP966 and 5’-AZA also significantly increased the mRNA expression of other tumor suppressors, including EGR1, PTEN, STAT1, and immune stimulatory receptor CD40 (**Figures 5B, C**). We also detected an increase in mRNA expressions of cell cycle related genes, such as *CCNB1*, *CDK1*, and *TSC1*, and transcriptional factors, such as C-Jun and C-Myc, upon RGFP966 and 5’-AZA treatment (**Table 2**). Therefore, change of chromosomal modifications in PDFS cells by HDAC3 inhibition and DNA demethylation led to activation of multiple tumor suppressors, resulting in growth suppression.

**Table 2:** Changes in gene expression changes by 5’-AZA and RGFP966 treatment.

Genes	Category	DMSO (Mean ± SD)	5’-AZA (Mean ± SD)	RGFP966 (Mean ± SD)	5’-AZA + RGFP966 (Mean ± SD)
**CCNB1**	Cell cycle	1.001 ± 0.071	1.732 ± 0.0484	1.194 ± 0.081	0.930 ± 0.0169
**CDK1**	Cell cycle	1.001 ± 0.030	0.396 ± 0.257	1.356 ± 0.113	1.051 ± 0.116
**TSC1**	Cell cycle	1.001 ± 0.037	1.327 ± 0.068	3.118 ± 0.532	1.791 ± 0.129
**EGR1**	TranscriptionFactor	1.340 ± 0.088	3.977 ± 0.097	0.884 ± 0.062	5.612 ± 0.077
**C-JUN**	TranscriptionFactor	1.038 ± 0.394	1.383 ± 0.049	2.322 ± 0.094	2.223 ± 0.072
**STAT1**	TranscriptionFactor	0.915 ± 0.001	1.920 ± 0.646	162.579 ± 14.801	698.954 ± 52.707
**C-MYC**	TranscriptionFactor	1.000 ± 0.014	2.604 ± 0.106	0.718 ± 0.069	2.074 ± 0.089
**P16^Ink4a^**	CDK inhibitor	1.019 ± 0.247	3.417 ± 1.221	9.314 ± 3.570	18.485 ± 1.278
**CD40**	Membrane Receptor	1.264 ± 0.175	559.435 ± 31.045	9.534 ± 2.226	1762.570 ± 72.253
**PTEN**	Tumor suppressor	1.001 ± 0.053	3.473 ± 0.054	2.469 ± 0.101	8.676 ± 0.051
**IL-6**	Cytokine	1.008 ± 0.177	2.353 ± 0.055	3.730 ± 0.265	4.820 ± 0.0189
**E2F1**	P16 downstream	1.000 ± 0.0271	3.273 ± 0.280	4.484 ± 0.478	7.880 ± 1.051

## Discussion

In this study, we investigated the expression of Class I HDAC family members in human NFPAs. We found a 40-60 fold elevation of the expression of HDAC2 and HDAC3 mRNA in NFPAs compared to normal pituitaries, suggesting their involvement in the pathogenesis of NFPAs. Increased mRNA expression of Class I HDAC members was also found in a limited number of hormone-secreting human pituitary adenomas, although no statistically significant difference was observed between hormone-secreting pituitary tumors and normal pituitaries. Further studies with a large number of such tumors are needed to verify this observation. Histone modification and abnormal expression of HDAC family members have been shown to be involved in the pathogenesis of many different human cancers. HDAC3 expression is not only elevated in these cancers, but is also often associated with cancer cell dedifferentiation (12, 17) and overall survival rate of patients (35, 36). However, the role of HDAC family members in the pathogenesis of human pituitary adenomas, a non-malignant neoplasm, has not been previously studied to our knowledge. Our study revealed the involvement of histone deacetylation in the pathogenesis of human NFPAs for the first time and provides a rationale for pursuing HDAC suppression as a possible therapeutic target.

In our study, treatment of PDFS cells with the HDAC3 inhibitor RGFP966 resulted in a 70% suppression of cell proliferation at 96 hours. Consistently, HDACs have been shown to control cell growth, differentiation, and apoptosis by regulating histone modification and acetylation of transcription factors including as p53 and E2F (37, 38). Multiple mechanisms have been revealed for HDAC3-mediated growth regulation. In colon cancer cells, silencing of HDAC3 expression by RNA interference (RNAi) resulted in growth suppression, accompanied by increased expression of p21 and apoptosis (39). In acute myeloid leukemia (AML), oncogenic protein c-myc recruits HDAC3 to form a suppressive complex binding to the promoter of miR-451 gene, inhibiting the expression of this tumor suppressive microRNA (40). Wells et al. showed that in cutaneous T cell lymphoma cells, HDAC3 was associated with chromatin around DNA replication forks; and inhibition of HDAC3 significantly reduced DNA replication, disrupting cycling of tumor cells (41). Moreover, all Class 1 HDACs, including HDAC2 and HDAC3, are known to down-regulate p53 by deacetylation at KK373/K382, reducing the binding of p53 to the promoters of its downstream targets such as p21 (42, 43). Consistent with these results, we have shown here that HDAC3 inhibition led to activation of several tumor suppressors, including EGR1, PTEN, STAT1, all downstream targets of p53. In addition, suppression of HDAC3 also resulted in the upregulation of MEG3, a lncRNA tumor suppressor specifically silenced in NFPAs, as well as components in the Rb signaling pathway such as P16 and E2F1, both of which have been suggested to be involved in the pathogenesis of NFPAs. In addition, epigenetic regulation of protein expression of P16, PTEN, and STAT1 has been well documented (44–46). As deacetylation and demethylation are global events, several studies have explored the epigenomics of pituitary adenomas, as summarized in a few excellent recent reviews (47, 48). Our results are consistent with these global transcriptomic analyses. Taken together, our data strongly suggest a role of HDAC3 in the development of NFPAs.

Histone modifications and DNA methylations are two important layers of epigenetic regulation and are often connected. There are many studies exploring the involvement of DNA methylations in pituitary adenomas, but substantial investigations of histone modification are yet to be done in pituitary tumors. After we found the increase expression of HADC2/3 in these tumors and observed changes in gene expression by the inhibitor RGF966, it is logical to explore the combined effect of RGP966 and 5’-AZA so we would see the corporative or synergistical functions of histone modifications and DNA methylations in pituitary tumors. Indeed, the combination of HDAC3 inhibitor RGFP966 with a DNA demethylation reagent, 5-aza-2-deoxycytidine, synergistically enhanced the expression of several tumor suppressors such as p16, PTEN, STAT1, CD40, and large non-coding RNA MEG3, accompanied by growth suppression. These data further emphasize the importance of epigenetic abnormalities in human NFPAs. A future study should investigate the combined effects of histone modification and DNA demethylation on global transcriptomic changes to understand the mechanisms by which histone modification and DNA demethylation regulate tumor growth. Because of the pivotal roles of HDACs in cancers, they have become targets for the development of novel cancer therapies. Indeed, Class 1 HDACs are targets for several cancer treatment drugs currently in use (49, 50). Some recently developed HDAC inhibitors have shown promising results in preclinical studies, either working alone (51–55) or in combination with other compounds (56, 57). In addition, it has been reported that suppression of HDAC3 sensitized cancer cells that had developed resistance to chemotherapies (58, 59). These data, together with our data reported here, suggest that HDAC3 is a potential target for use in therapy of NFPAs. Further studies will be needed to confirm our findings, explore the mechanisms in detail, and validate our findings in animal models and other pre-clinical models.

## Data Availability Statement

The original contributions presented in the study are included in the article/**Supplementary Material**. Further inquiries can be directed to the corresponding author.

## Author Contributions

WZ: performing experiments, manuscript and figure preparations. XJ, KW, JM, EB, BW: performing experiments. E.TH-W, BS: critical clinical input and discussion. YZ, AK: study design, organization, and supervision. RS: critical scientific input and discussion, manuscript preparation. XZ: study design, organization, supervision, manuscript and figure preparation. All authors contributed to the article and approved the submitted version.

## Funding

This work was supported in part by the National Institutes of Health (R01 CA193520 to AK and RS), and the Jarislowsky Foundation. None of the funding sources was involved in study design; in the collection, analysis and interpretation of data; in the writing of the report; and in the decision to submit the article for publication.

## Conflict of Interest

The authors declare that the research was conducted in the absence of any commercial or financial relationships that could be construed as a potential conflict of interest.

## Publisher’s Note

All claims expressed in this article are solely those of the authors and do not necessarily represent those of their affiliated organizations, or those of the publisher, the editors and the reviewers. Any product that may be evaluated in this article, or claim that may be made by its manufacturer, is not guaranteed or endorsed by the publisher.

## Supplementary Material

Supplementary Table 1 Primers. *MGH-PGA Prime Bank: https://pga.mgh.harvard.edu/primerbank/.
