# Supplementary material for: High Histone Deacetylase 2/3 Expression in Non-Functioning Pituitary Tumors
Source: Front Oncol. 2022 May 13;12:875122. doi: 10.3389/fonc.2022.875122 (PMC9136140; doi:10.3389/fonc.2022.875122)
Supplement: Supplementary Table 1 — Primers. *MGH-PGA Prime Bank: https://pga.mgh.harvard.edu/primerbank/. [file Table_1.docx]

**Supplementary data**

**Table S1. Primers**

| **Gene** | **Gene Bank ID** | Primers(F/R 5’-3’) | Product Size (bp) | Ref |
| --- | --- | --- | --- | --- |
| MEG3 | AF151783 | F:CTGCTGCCCATCTACACCTCAC;  R:CCTCTTCATCCTTTGCCATCCTGG | 100 | * |
| DNMT1 | NM_001130823 | F:AGAACGGTGCTCATGCTTACA;  R:CTCTACGGGCTTCACTTCTTG | 81 | * |
| DNMT3A | NM_175629 | F:CCGATGCTGGGGACAAGAAT;  R:CCCGTCATCCACCAAGACAC | 151 | * |
| DNMT3B | NM_001207055 | F:ACCTCGTGTGGGGAAAGATCA;  R:CCATCGCCAAACCACTGGA | 121 | * |
| HDAC1 | NM_004964 | F:CGCCCTCACAAAGCCAATG;  R:CTGCTTGCTGTACTCCGACA | 108 | * |
| HDAC2 | NM_001527 | F:ATGGCGTACAGTCAAGGAGG;  R:TGCGGATTCTATGAGGCTTCA | 112 | * |
| HDAC3 | NM_003883 | F:TCTGGCTTCTGCTATGTCAACG;  R:CCCGGTCAGTGAGGTAGAAAG | 136 | * |
| CCNB1 | AY338491 | F:AATGAAATTCAGGTTGTTGCAGGAG;  R:CATGGCAGTGACACCAACCAG | 197 | S1 |
| CDK1 | NM_001786 | F:AAACTACAGGTCAAGTGGTAGCC;  R:TCCTGCATAAGCACATCCTGA | 147 | S2 |
| TSC1 | NM_000368 | F:TAGGCTGGAGGGACTGTGAG;  R:CCTGGTGTCTTTCATGGTCA | 111 | S3 |
| EGR1 | NM_001964 | F:GGTCAGTGGCCTAGTGAGC;  R:GTGCCGCTGAGTAAATGGGA | 149 | * |
| C-Jun | NM_002228 | F:TGACTGCAAAGATGGAAACG;  R:CAGGGTCATGCTCTGTTTCA | 118 | S4 |
| STAT1 | NM_007315 | F:CTAGTGGAGTGGAAGCGGAG;  R:CACCACAAACGAGCTCTGAA | 251 | S5 |
| C-MYC | NM_002467 | F:CGTCTCCACACATCAGCACAA;  R:TGTTGGCAGCAGGATAGTCCTT | 95 | S6 |
| P16^lnk4a^ | NM_000077 | F:CCCAACGCACCGAATAGTTA;  R:ACCAGCGTGTCCAGGAAG | 172 | S7 |
| CD40 | NM_000074 | F:TTGGGGTCAAGCAGATTGCTA;  R:GCAGATGACACATTGGAGAAGA | 85 | * |
| PTEN | NM_000314 | F:TTTGAAGACCATAACCCACCAC;  R:ATTACACCAGTTCGTCCCTTTC | 134 | * |
| IL-6 | NM_000600 | F:CCAGCTATGAACTCCTTCTC;  R:GCTTGTTCCTCACATCTCTC | 425 | S8 |
| E2F1 | NM_00522 | F:CATCCCAGGAGGTCACTTCTG;  R:GACAACAGCGGTTCTTGCTC | 145 | * |
| GAPDH | NM_002046 | F:CTGGGCTACACTGAGCACC;  R:AAGTGGTCGTTGAGGGCAATG | 101 | * |

*:MGH-PGA Prime Bank: https://pga.mgh.harvard.edu/primerbank/

**Supplementary references:**

S1. Shah, Y.M. and B.G. Rowan, The Src kinase pathway promotes tamoxifen agonist action in Ishikawa endometrial cells through phosphorylation-dependent stabilization of estrogen receptor (alpha) promoter interaction and elevated steroid receptor coactivator 1 activity. Mol Endocrinol, 2005. **19**(3): p. 732-48.

S2. Glozak, M.A. and E. Seto, Histone deacetylases and cancer. Oncogene, 2007. **26**(37): p. 5420-32.

S3. Yamakoshi, K., et al., Real-time in vivo imaging of p16Ink4a reveals cross talk with p53. J Cell Biol, 2009. **186**(3): p. 393-407.

S4. Wang, L., et al., Resistance exercise enhances the molecular signaling of mitochondrial biogenesis induced by endurance exercise in human skeletal muscle. J Appl Physiol (1985), 2011. **111**(5): p. 1335-44.

S5. Kim, A.R., et al., Functional Selectivity in Cytokine Signaling Revealed Through a Pathogenic EPO Mutation. Cell, 2017. **168**(6): p. 1053-1064 e15.

S6. Tao, Y.F., et al., Molecular mechanism of G1 arrest and cellular senescence induced by LEE011, a novel CDK4/CDK6 inhibitor, in leukemia cells. Cancer Cell Int, 2017. **17**: p. 35.

S7. Li, L., et al., Transcriptional Regulation of the Warburg Effect in Cancer by SIX1. Cancer Cell, 2018. **33**(3): p. 368-385 e7.

S8. Weigert, C., et al., Upregulation of IL-6 mRNA by IL-6 in skeletal muscle cells: role of IL-6 mRNA stabilization and Ca2+-dependent mechanisms. Am J Physiol Cell Physiol, 2007. **293**(3): p. C1139-47.
